# Practical dose delivery verification of craniospinal IMRT

**DOI:** 10.1120/jacmp.v16i6.5481

**Published:** 2015-11-08

**Authors:** Young K Lee, Anthony T Kim, Peiying Zhao, Aliaksandr Karotki

**Affiliations:** ^1^ Department of Medical Physics Odette Cancer Centre, Sunnybrook Health Sciences Centre Toronto ON Canada; ^2^ Department of Radiation Oncology University of Toronto Toronto ON Canada

**Keywords:** craniospinal irradiation, CSI, IMRT, pretreatment verification, whole CNS

## Abstract

Craniospinal irradiation (CSI) using IMRT allows dose sparing to organs‐at‐risk (OAR) whilst conforming the dose to the target volume. Due to the complexity of treatment involving several isocenters, the dose distribution created by the inverse‐planned segmented fields must be verified prior to treatment. We propose and test methods to verify dose delivery using commonly available dosimetry equipment for commissioning and routine patient‐specific dose verification of craniospinal IMRT. Ten patients treated with conventional CSI were retrospectively planned with a 3‐isocenter (cranial, upper spine, and lower spine) IMRT technique. The isocenters were placed 25–27 cm away from each other in the longitudinal direction but in the same lateral and anterioposterior positions. The planning target volume (PTV) was defined as the brain with a 0.5 cm margin and spinal canal with a 1.0 cm margin. The plans were prescribed to 36 Gy in 20 fractions to the PTV mean dose. Eleven beams (five cranial, three upper spine, and three lower spine) were optimized simultaneously. The dose delivered by the IMRT plans was then recalculated on several different phantoms and measured using the following methods: 1) ionization chamber inserted in a cylindrical phantom, positioned in the junction regions between cranial/upper‐spine isocenters and upper‐/lower‐spine isocenters; 2) MapCHECK centered in the overlap regions; and 3) ArcCHECK measurement with beams from each isocenter. For 1) ±3% dose difference and for 2) and 3) ≥95% of measured points with a γ‐index <1 for 3% dose difference and 3 mm distance‐to‐agreement were deemed clinically acceptable. The median (range) planned to measured dose differences for the ionization chamber is 0.4% (−1.5% to 3.0%) for the cranial/upper‐spine field and 1.8% (−0.8% to 2.6%) for the upper‐/lower‐spine field overlap region. The median (range) percentage of MapCHECK diodes with a γ index <1 for 3%/3 mm criterion is 98.0% (95.3% to 99.7%) for the cranial/upper‐spine and 97.3% (95.0% to 99.6%) for the upper‐/lower‐spine field overlap regions. The median (range) percentage of ArcCHECK diodes with a γ index <1 for 3%/3 mm criterion is 99.7% (97.1% to 100%). Three different methods of verifying craniospinal IMRT were compared and tested. All techniques offer different benefits and together can be used for 1) commissioning the treatment technique and, separately, for 2) patient‐specific pretreatment verification measurements.

PACS number: 87.55.km

## I. INTRODUCTION

Craniospinal irradiation (CSI) using intensity‐modulated radiation therapy (IMRT) allows for homogeneous and conformal dose distribution throughout the target volume while reducing dose to the surrounding tissue.^1^, ^2^, ^3^, ^4^, ^5^, ^6^, ^7^ It differs from the conventional techniques that rely on single plane matched junction in the neck region with a "gapped" junction in the spine, in which very high‐ and low‐dose regions are produced in the treatment volume.

Although craniospinal IMRT has these benefits, the segmented and the overlapping fields created by IMRT and the resulting dose distribution are complex. The junction regions are of particular interest due to being very uncommon for IMRT based treatments. Therefore it is important to verify the dose delivery for commissioning of the new technique, as well as for pretreatment patient dosimetric verification. Many centers still treat CSI using conventional methods in both supine and prone positions, and as the number of cases per center is relatively small, it would be difficult to move onto a new CSI technique easily. Furthermore, dosimetric equipment is designed to measure relatively small treatment volumes, such as the prostate, and no methodological studies exist on how to verify large‐volume treatments such as CSI, which are relatively uncommon in radiation oncology. The purpose of this paper is to describe and compare various methods for verifying CSI IMRT dose delivery using commonly available dosimetry equipment for commissioning and routine patient‐specific dose verification.

## II. MATERIALS AND METHODS

### A. Treatment planning methodology

**Figure 1:**
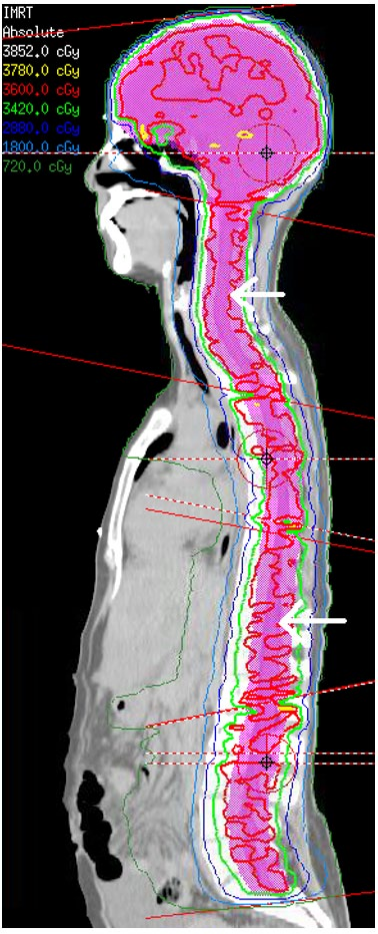
Typical dose distribution for a CSI‐IMRT plan. Arrows indicate the approximate field overlapped regions measured with ionization chamber (both arrows) and MapCHECK (bottom arrow only). The black circles shown in the image indicate the isocenter positions (from top to bottom are cranial, upper‐, and lower‐spine isocenters, respectively). PTV is shown in pink colourwash and the isodoses are specified in the legend.

Ten patients treated with conventional CSI were retrospectively planned with a three‐isocenter (cranial, upper spine, and lower spine) IMRT technique using Philips Pinnacle v9.2 (Philips Healthcare, Andover, MA). The planning target volume (PTV) was defined as the combined volume of brain with 0.5 cm uniform margin and spinal canal localized to approximately S3 level with a 1.0 cm uniform margin. The isocenters (see Fig. 1) were placed 25–27 cm away from each other. With the maximum field size of 40 cm, that resulted in overlapped regions of 13–15 cm. The plans were prescribed to 36 Gy in 20 fractions to the mean dose to the PTV. All patient‐treatment plans were created and delivered using the Elekta Synergy (Crawley, West Sussex, UK) 6 MV beams. Eleven beams were used in the treatment plan: five beams at cranial isocenter (gantry angles 0°, 40°, 95°, 265°, and 320°), three beams at the upper‐spine isocenter, and three beams at the lower‐spine isocenter (spine gantry angles 145°, 180°, and 215°). Collimator angles were chosen so that the multileaf collimators can best conform to the PTV for all cranial and spinal fields.

**Figure 2:**
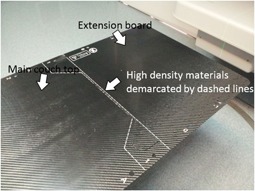
Schematic showing the "high density" region of the couch with the extension board attached.

Placing the cranial fields requires some care due to the construction of the Elekta couch top. The carbon fiber couch top is a single piece that is mounted to the couch pedestal and can be lengthened with the addition of a head extension board (see Fig. 2). Since CSI patient volumes are long, the use of an extension board is often necessary, particularly for adult patients. At the end of the main couch, where the extension board clips on, there are high‐density materials embedded into the carbon fiber to maintain the rigidity of the extension board mount. This high‐density region is demarcated by white dashed lines on the couch and extension board. No posterior or posterior‐oblique cranial beams were used, to avoid the high‐density region in the Elekta couch top.

All beams were optimized simultaneously with a high weight placed on the PTV with lower weight placed on the organs‐at‐risk (OAR) objectives. The inverse planning objectives for the OARs were subsequently increased to minimize the dose to the OARs. The plans were assumed to have reached their potential when OAR doses could not be decreased without compromising on PTV coverage. No special emphasis was placed on the junction regions and the homogeneous dose distribution throughout the junctions was achieved automatically by the inverse planning algorithm as part of the inverse‐planning objectives used for the PTV. Figure 1 shows a typical dose distribution of an IMRT plan.

#### A.1 Method 1: Point measurement (ionization chamber)

**Figure 3:**
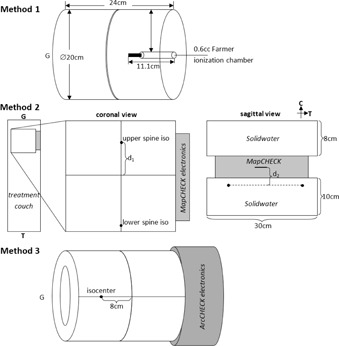
Schematic of measurement setup methods. Method 1 shows the cylindrical phantom with the inserted 0.6 cc Farmer ionization chamber. Method 2 shows MapCHECK position and orientation to measure dose distribution in the junction region created by the fields from two adjacent isocenters. For each patient, the measuring MapCHECK plane was placed such that it would best measure the dose from the junction region. For Method 3, ArcCHECK was positioned with the isocenter centered but 8 cm away in the longitudinal direction. G = gun, T = target, C = ceiling.

The IMRT plans were recalculated on the CT scan of an in‐house cylindrical water‐equivalent phantom with a centrally inserted 0.6 cm^3^ Farmer ionization chamber (see Fig. 3 Method 1). The couch attenuation was accounted for by adding a couch model in the treatment planning calculation. The position of the ionization chamber was selected such that it was in a relatively high and homogeneous dose region. Measurements were made in both the cranial/upper‐spine and upper‐/lower‐spine field overlapping regions. The general regions where the measurements were taken are indicated by the arrows in Fig. 1. A measured‐to‐planned ionization chamber dose difference of ±3% was considered clinically acceptable.

#### A.2 Method 2: Two‐dimensional distribution (MapCHECK)

The IMRT plans were recalculated and measured on a MapCHECK diode array (Sun Nuclear Corp., Melbourne, FL). The setup is shown in Fig. 3 Method 2. The MapCHECK measurement was made for both cranial/upper‐spine and the upper‐/lower‐spine overlap regions. Positioning MapCHECK in a junction area between brain and upper‐spine isocenters using the "planned geometry" would irradiate the device electronics due to lateral cranial fields. Therefore, to test the cranial/upper‐spine field overlap region, nominal gantry (0°) and planned collimator angles were used for all cranial and upper‐spine beams, and a composite plan was calculated on MapCHECK and compared to measurement (Fig. 3 Method 2A). For the upper‐/lower‐spine field overlap region, the beams were recalculated on MapCHECK with 180° added to gantry angles to irradiate the device anteriorly. The radiologic depth of the MapCHECK diodes is 2 cm from the surface of the device. With 8 cm of solid water placed on top of the MapCHECK, the measuring plane was in a water‐equivalent depth of 10 cm. Shifts were made such that the measuring plane was in a high‐dose region of interest (see Fig. 3 Method 2).

Percent measured points with γ index <1 for dose/distance‐to‐agreement criteria of 3%/3 mm, 3%/ 2 mm, 2%/3 mm, and 2%/2 mm were tested.^(8)^ Greater than 95% of measured points meeting the criterion of 3% absolute dose difference and 3 mm distance‐to‐agreement was deemed clinically acceptable.

#### A.3 Method 3: Three‐dimensional distribution (ArcCHECK)

The fields from each isocenter were recalculated and measured on an ArcCHECK diode array (Sun Nuclear Corp.) as shown in Fig. 3 Method 3. Fields from each isocenter were measured separately with a couch shift of 8 cm in the longitudinal direction to avoid irradiating the device electronics. The DICOM parameter ImagePositionPatient in the dose files was edited to account for 8 cm longitudinal shift to maximize the analyzed volume in the ArcCHECK software.

Percent measured points with γ index <1 for dose/distance‐to‐agreement criteria of 3%/3 mm, 3%/2 mm, 2%/3 mm, and 2%/2 mm were tested for plans calculated at each isocenter. Greater than 95% of measured points meeting the criterion of 3% absolute dose difference and 3 mm distance‐to‐agreement for all measurements made for each plan (i.e., measurements made at each of the three isocenters) were deemed clinically acceptable.

## III. RESULTS

### A.1 Method 1: Point measurement (ionization chamber)

**Figure 4:**
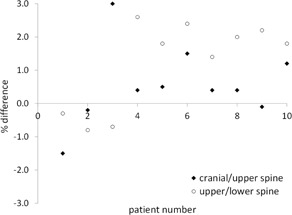
Percent difference between calculated and planned dose to the ionization chamber in a water‐equivalent cylindrical phantom. A positive number indicates planned dose is greater than the measured dose.

The median (range) planned to measured dose differences for the ionization chamber are 0.4% (−1.5% to 3.0%) in the cranial/upper‐spine and 1.8% (−0.8% to 2.6%) for the upper‐/lower‐spine field overlap regions (Fig. 4). For 14 of 20 measurements, the planned dose is greater than the measured dose. The largest difference is in Patient #3 cranial/upper‐spine overlap region, where the planned dose is 3.0% higher than the measured dose. For all junctions, the measured dose does not have clinically relevant differences to the planned dose.

### A.2 Method 2: Two‐dimensional distribution (MapCHECK)

The MapCHECK results for all patients are presented in Table 1. The median (range) percentage of diode measurements with a γ index <1 for 3%/3 mm criterion is 98.0% (95.3% to 99.7%) for cranial/upper and 97.3% (95.0% to 99.6%) for upper‐/lower‐spine junction. All the results are within acceptable levels based on our center's clinical practice. In the cranial/upper‐spine junction, for 3%/2 mm, 2%/3 mm, and 2%/2 mm criteria, 5 of 10, 7 of 10, and 2 of 10 data, respectively, show >95%; and 7 of 10, 10 of 10, and 5 of 10 data, respectively, show >90% measured points with γ index <1. In the upper‐/lower‐spine junction, for 3%/2 mm, 2%/3 mm, and 2%/2 mm criteria, 4 of 10, 4 of 10 and 1 of 10 data, respectively, show >95%, and 7 of 10, 10 of 10, and 4 of 10 data, respectively, show >90% measured points with γ index <1. The measured dose was in general lower than the planned dose for all patients. The measurement for Patient #2 had the lowest result in the cranial/upper‐spine junction and Patient #1 had the lowest result in the γ‐analysis pass rate.

**Table 1:** MapCHECK measured‐to‐planned two‐dimensional dose distribution comparison using the γ‐analysis. The results for the cranial/upper‐spine field overlap region is shown on the left and upper‐/lower‐spine field overlap region is shown on the right. All MapCHECK results in % for the γ‐analysis

	*Cranial/Upper Spine*				*Upper/Lower Spine*			
*Pt. #*	3%/3mm	3%/2mm	2%/3mm	2%/2mm	3%/3mm	3%/2mm	2%/3mm	2%/2mm
1	99.2	98.4	95.9	94.3	95.0	89.2	90.6	83.1
2	95.3	89.3	90.9	82.2	95.6	88.8	94.4	86.0
3	97.5	92.9	95.4	86.8	99.2	98.4	98.0	96.7
4	98.6	95.9	96.4	88.2	96.9	89.9	94.1	86.1
5	98.2	94.1	96.7	91.1	96.7	94.2	94.7	87.7
6	95.6	89.1	91.6	81.9	96.2	92.5	92.5	87.0
7	97.1	89.1	92.6	85.4	98.4	96.4	97.6	92.9
8	99.4	98.0	98.5	94.8	99.6	97.5	97.5	93.4
9	97.8	97.5	97.8	95.9	97.7	94.4	95.9	90.6
10	99.7	97.6	98.0	96.0	98.3	97.8	94.8	87.8

### A.3 Method 3: Three‐dimensional distribution (ArcCHECK)

**Figure 5:**
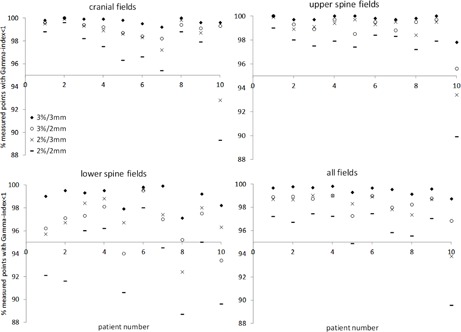
ArcCHECK results of measured‐to‐planned three‐dimensional dose distribution comparison using the γ‐analysis for the 10 patient cases are plotted. Fields from each isocenter are shown separately (top left, top right, and bottom left) and the total diode measurement results for the plan are also plotted (bottom right).

The ArcCHECK results for each isocenter from the 10 patient data are shown in Fig. 5. All the results are within acceptable levels based on our center's clinical practice. Greater than 95% of measured points with a γ index <1 criterion of 3%/2 mm, 2%/3 mm, and 2%/2 mm are seen for 28 of 30, 27 of 30, and 22 of 30 cases, respectively. Cranial and upper‐spine isocenter measurements had higher γ‐analysis pass rates than lower‐spine isocenter measurements with tighter criteria (3%/2 mm, 2%/3 mm, and 2%/2 mm). The measurement for Patient #10 resulted in the worst γ‐analysis pass rate.

## IV. DISCUSSION

CSI with IMRT results in superior dose distribution compared to conventional techniques. But it comes at a price of delivering the dose with complicated inverse‐planned segments using multiple isocenters. As such, the pretreatment dose delivery verification is needed in the context of both commissioning the technique, as well patient‐specific pretreatment dose delivery verification. Unfortunately, dosimetric equipment used for patient treatment verification, such as measurement arrays used in this study, is designed to measure dose in relatively small treatment volumes. Junctions constitute a particular problem. Since most IMRT (non‐CSI) treatments do not involve junctions, the methodology for verifying dose delivery in the junction regions planed with inverse‐planning algorithm is not well developed. Dose measurement of large volumes is usually done without a procedure in place, and therefore can cause delays in treatment start date as they are difficult to analyze quickly as required in clinical practice. In this study, we demonstrated several methods of verifying IMRT dose delivery for treatment plans involving three isocenters with overlapping fields. Note that it can be extended to any treatment site with multiple isocenters.

Several previous studies have discussed CSI dose verification but most of them tested the principle of their planning methodology and the resulting measurements have not been thoroughly explained.^1^, ^2^, ^3^, ^4^, ^5^, ^6^ Most of the studies have used film measurements, which were difficult to set up and analyze and typically required confirmation of absolute dose with another measurement. Seppälä et al.^(1)^ used a single film to verify dose delivery. A single profile from the film measurement was presented and relative dose comparison was made.^(1)^ Cao et al.^(3)^ conducted EPIDose^(9)^ and film measurements. Their IMRT plans passed the EPIDose test with a 3% dose and 3 mm positional tolerances. However, EPIDose usage with such large fields is not well documented and has not been proven. Wang et al.^(4)^ used a 2D array system to measure dose distribution in the junction region for nine patients and reported pass rate of 90% when using 3%/3 mm criterion. It was not clear whether this was a clinically acceptable level for their center. Furthermore, the setup methods were not described and only the spine‐spine junction regions had been tested. Studenski et al.^(5)^ presented a single case study where a comparison was made between single plane film measurement and planned dose distribution presented as a percent difference. This showed a large difference in dose of >20% in small regions in the neck, which was accepted by the authors in the junction region between conventional cranial and IMRT spine fields. They also did MapCHECK measurements in the junction regions and observed pass rate of less than 50% for 3%/3 mm criterion when absolute dose was compared. They also had tested composite plans using the nominal gantry angle and concluded that the low pass rates were due to the low resolution of the MapCHECK diodes. We have attempted to simulate the planned field positions and dose distribution as much as possible by only flipping the gantry angles such that the fields always entered anterior and anterior‐oblique to the MapCHECK for the spine junction. Furthermore, we chose to place the MapCHECK center such that it was in relatively high‐dose plane for measurement, where the dose would be most relevant. The difference in our and the study by Studenski and colleagues may be a reflection of setup differences. The solid water/ionization chamber result from the study by Studenski et al. was comparable to our cylindrical phantom/ionization chamber results.

The results shown in this study were within the clinical levels expected for small‐volume IMRT pretreatment verification (γ index criterion of 3%/3 mm had pass rates of over 95%).

There was no correlation between the results of the three methods that were presented here. This is most probably due to the different regions that were tested in the three methods.

The ionization chamber measurement is useful to confirm absolute dose in both junction regions. It is easy to set up, but only gives information at a single point. Ionization chamber measurements should most probably be used to complement other pretreatment verification methods or as a part of commissioning measurements. MapCHECK allows verification of dose delivery in a junction region in a plane as opposed to a point. We simulated and tested as similar to the planned geometry without irradiating the MapCHECK electronics. Both MapCHECK and ion chamber were used extensively during commissioning of the craniospinal IMRT at our institution and allowed us to gain confidence in the accuracy of the dose in the junction regions as calculated by Pinnacle. They are not used as part of the routine patient‐specific pretreatment dose verification. ArcCHECK allows extension of dose verification to 3D. As such it is the most powerful dose verification device with largest amount of information about dose delivery accuracy being provided. But it suffers from the same problem as MapCHECK (i.e., if placed in the junction region, the electronics will be irradiated). Therefore, we used it during commissioning phase to verify dose separately for each isocenter. And we continue using it in the same fashion for routine patient‐specific pretreatment dose verification.

## V. CONCLUSIONS

We have shown that large multiple‐isocentric IMRT dose distribution, such as the CSI IMRT, can be verified using commonly available dosimetry equipment. All three methods described in the paper — point measurement with ion chamber, 2D measurement with MapCHECK, and 3D measurement with ArcCHECK — have their own benefits and complement each other. All three methods proved to be useful during the commissioning phase of the craniospinal IMRT. The differences in measured‐to‐planned dose show that IMRT‐based CSI is deliverable and clinically acceptable for treatment. For patient‐specific pretreatment dose delivery verification, we settled on using ArcCHECK to verify dose separately for every isocenter.
